# Single site pacing through the anterior interventricular vein in a patient with a mechanic tricuspid valve

**Published:** 2009-05-15

**Authors:** Matteo Anselmino, Maria Cristina Marocco, Marcella Jorfida, Riccardo Massa

**Affiliations:** Cardiology Unit, Department of Medicine, University of Turin, Italy

**Keywords:** mechanical tricuspid valve, pacing, coronary sinus, anterior interventricular vein

## Abstract

Transvenous endocardial pacing through classical implantation of a pace/sensing lead in the right ventricle is strictly contraindicated in patients with a mechanical tricuspid valve. Usually permanent pacing is achieved by an epimyocardial surgical approach. We hereby describe the implantation of a single site left ventricle pacing lead in the anterior interventricular vein in a 60 year-old woman with symptomatic bradycardia, permanent atrial fibrillation, and mechanical tricuspid valve. The described use of left ventricle pacing through a coronary vein lead, in a patient with favorable venous anatomy, provided (through a minimal invasive approach) effective with a low and stable threshold.

## Case Report

A 60 year-old woman with permanent atrial fibrillation was referred to our University center for pacing system implantation due to symptomatic bradycardia.

Clinical history reported acute rheumatic fever in the childhood followed by, at 41 years of age, surgical mitral and aortic mechanic and tricuspid bioprosthetic valve replacements. Three years later degeneration of the tricuspid bioprosthesis, accompanied by grade IV regurgitation, required replacement with a new tricuspid mechanic valve (St. Jude n.31).

**Figure 1:**
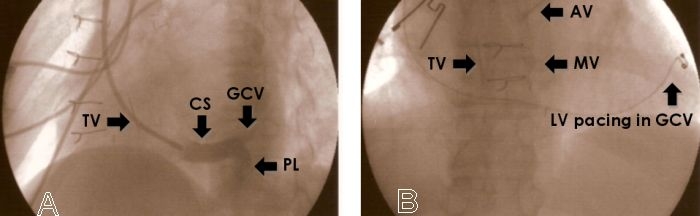
**A** (left side) - A 60 degree left anterior view showing coronary sinus venogram (coronary sinus, CS, posterolateral branch, PL, and anterior interventricular vein or great cardiac vein, GCV) and tricuspid valve, TV. **B** (right side) - An antero-posterior view showing distal course of the transvenous left ventricular, LV, pacing lead in the distal GCV; TV, mitral, MV, and aortic, AV, valves.

A guiding catheter was introduced and placed into the ostium of the coronary sinus via the left subclavian vein. Venography of the coronary veins was performed to establish detailed anatomy (Figure 1A). A bipolar endocardial pacing lead (Pacesetter Quicksite 1056T, St. Jude) was implanted in the anterior interventricular vein (Figure 1B).

Measurements at implantation reported pacing threshold 0.5 V at 0.4 ms, R wave sensing potential 20.7 mV, impedance 1160 ohms at 5 V. Electrocardiogram showed left ventricle pacing with right bundle branch block configuration. The lead was secured and connected to a single-chamber rate adaptive pacemaker (Sensia SESR01, Medtronic) placed in a subfascial prepectoral pocket and programmed to ventricular demand rate-responsive at 50 ppm. In day 2 after the implantation chest X-ray excluded lead dislodgement and controller based pacemaker measurements were stable. Six months later, the last available follow-up for this woman, all pacemaker parameters remained stable (threshold 0.75 V at 0.4 ms; R-wave sensing 19.1 mV; impedance 917 ohms at 5 V) in the absence of symptomatic arrhythmic events.

## Discussion

Transvenous endocardial pacing through classical implantation of a pace/sensing lead in the right ventricle is strictly contraindicated in patients with mechanical tricuspid valve due to the likely damage of the prosthetic valve and/or lead fracture at the tricuspid site. Usually, in this situation, permanent pacing is achieved by an epimyocardial surgical approach. Epimyocardial lead placement however has not proved as successful as endocardial lead placement due to the invasive nature of this approach when done with a larger incision and to increasing thresholds when performed in a minimally invasive manner [1].

In the afore described case the woman underwent transvenous placement of a left ventricular pacemaker via the coronary sinus given her firm refusal to undergo a new (the third) thoracotomy/sternotomy. The left ventricle pacing approach in patients with mechanical tricuspid valve replacement was first reported 14 years ago [2] but strongly limited by high frequencies of lead dislodgement and suboptimal pacing thresholds. Following this experience, few cases have been described in the worldwide literature [3,4].

In our experience the use of left ventricle pacing through a coronary vein lead, in a patient with prosthetic tricuspid valve and favorable vein anatomy, provided, through a minimal invasive approach, effective stimulation of the ventricle with low and stable threshold. Therefore, supported by the continuous implementation of new materials, left ventricular pacing may be considered as a feasible option for patients with mechanical tricuspid valves requiring permanent ventricular pacing (particularly in case of previous cardiothoracic surgery).
